# Incidence, diagnosis, treatment methods, and outcomes of clinically suspected venous thromboembolic disease in patients with COVID-19 in a quaternary hospital in Brazil

**DOI:** 10.1590/1677-5449.200203

**Published:** 2021-06-11

**Authors:** Marcela Juliano Silva Cunha, Carlos Augusto Ventura Pinto, João Carlos de Campos Guerra, Adriano Tachibana, Maria Fernanda Cassino Portugal, Leonardo José Rolim Ferraz, Nelson Wolosker

**Affiliations:** 1 Hospital Israelita Albert Einstein – HIAE, São Paulo, SP, Brasil.; 2 Universidade de São Paulo – USP, Faculdade de Medicina, São Paulo, SP, Brasil.

**Keywords:** thrombosis, thromboembolism, pulmonary embolism, coronavirus disease-19, death, drug therapy, trombose, tromboembolismo venoso, embolia pulmonar, COVID-19, óbito, tratamento farmacológico

## Abstract

**Background:**

Prothrombotic states have been associated with viral infections and the novel Sars-COV-2 infection has been associated with elevated D-dimer levels, although no causal relation has been clearly established.

**Objectives:**

This study presents an epidemiological analysis of manifest VTE episodes in a group of patients hospitalized because of COVID-19.

**Methods:**

Medical records of patients who presented symptomatic deep vein thrombosis and/or pulmonary embolism in concomitance with confirmed COVID-19 were retrospectively studied. Demographic characteristics, prevalence of VTE, site of occurrence, D-dimer variation over time, management, and outcomes were analyzed.

**Results:**

During the study period, 484 confirmed cases of COVID-19 were admitted, 64 of which displayed VTE symptoms and 13 of which had confirmed symptomatic VTE(2.68% of total sample and 20.31% of symptomatic cases). Most cases (76.92%) occurred in intensive care. On the day attributed to VTE onset, D-dimer levels were over 3,000 ng/mL in 8 (80%) patients, a significant increase from baseline admission levels (*p* < 0.05). A significant decrease was also observed in D-dimer values at hospital discharge (*p* < 0.05). All patients received pharmacological thromboprophylaxis and/or anticoagulation as indicated. Two deaths occurred during the study, both patients with severe comorbidities. At the end of our study protocol, nine patients had been discharged and two remained hospitalized, but had no signs of VTE worsening.

**Conclusions:**

VTE prevalence in hospitalized COVID-19 patients was 2.7%, and higher in intensive care units. Early institution of prophylaxis and immediate full anticoagulation when VTE is diagnosed should be the goals of those who treat this kind of patient.

## Resumo

**Contexto:**

Os estados pró-trombóticos têm sido associados a infecções virais. A nova infecção pela síndrome respiratória aguda grave do coronavírus 2 (SARS-CoV-2) sabidamente eleva os níveis de D-dímero, embora a relação causal não tenha sido bem estabelecida.

**Objetivos:**

Este estudo apresenta uma análise epidemiológica de episódios sintomáticos de tromboembolismo em um grupo de pacientes hospitalizados pela doença do novo coronavírus (COVID-19).

**Métodos:**

Foi realizada uma revisão retrospectiva de prontuários de pacientes internados por COVID-19 que apresentaram trombose venosa profunda e/ou embolia pulmonar sintomáticas. Foram avaliados os dados demográficos, a prevalência de tromboembolismo, a variação do D-dímero ao longo do tempo, o manejo e os desfechos.

**Resultados:**

Dos 484 casos confirmados de COVID-19 admitidos entre março e julho de 2020, 64 apresentaram sintomas de tromboembolismo, que foram investigados, e 13 tiveram tromboembolismo confirmado (2,68% do total e 20,31% dos sintomáticos). A maioria dos casos ocorreu em regime de terapia intensiva (76,92%). Houve um aumento significativo no número de pacientes com D-dímero acima de 3.000 ng/mL no dia atribuído ao diagnóstico de tromboembolismo com relação aos níveis do momento da admissão (80%, p < 0,05).Uma queda significativa de pacientes nesse limiar também foi observada no momento da alta (p < 0,05). Todos os pacientes receberam tromboprofilaxia ou anticoagulação conforme indicado. Houve dois óbitos na amostra, ambos pacientes com comorbidades severas. Ao fim do protocolo, nove pacientes receberam alta e dois permaneceram hospitalizados, mas sem sinais de piora.

**Conclusões:**

A prevalência de tromboembolismo em pacientes hospitalizados por COVID-19 foi de 2,7%, sendo mais frequente em regime de terapia intensiva. A instituição precoce de profilaxia e anticoagulação imediata ao diagnóstico é primordial nesse grupo de pacientes.

## INTRODUCTION

Venous thromboembolism (VTE) is a serious health concern, affecting up to 1 in 1,000 adults worldwide each year. Epidemiological studies have shown that prior to the institution of consistent in-hospital prophylaxis, 55 to 60% of all VTE cases were related to hospitalizations, being diagnosed either while still hospitalized or during the first 90 days after the patient’s discharge.^1^ Deep venous thrombosis (DVT) is the greatest cause of preventable in-hospital deaths, with an annual mortality rate in Brazil of 2.09 per 100,000 inhabitants.^2^^,^ 3

Viral outbreaks can lead to hypercoagulable states and deaths due to VTE, strokes and hemorrhagic disorders.^4^ In the last months of 2019, a new coronavirus (severe acute respiratory syndrome coronavirus 2 or SARS-CoV-2) was identified as the agent of a pneumonia outbreak that quickly spread, reaching pandemic status and being named COVID-19.^5^

The Sars-COV-2 infection has been associated with elevated levels of D-dimer (DD), although no causal relation has been clearly established.^6^ Further studies are needed to determine if there is a positive significant association. Comprehensive knowledge of the relationship between COVID-19 and VTE enables better decision-making concerning thrombotic and hemorrhagic risks.

Little is known about the pathogenic mechanism through which SARS-CoV-2 is able to trigger the chain reaction of immunologic, inflammatory, and coagulation responses in human patients.

To date, studies that have analyzed the clinical and epidemiological characteristics of patients with VTE related to infection by SARS-CoV-2 have reported conflicting results.^7^^-^ 11

We present an epidemiological analysis of COVID-19 patients who were admitted to a quaternary hospital in Brazil and presented clinically symptomatic thromboembolic events with an analysis of the diagnostic methods, management, and outcomes of these cases.

## METHODS

This is a retrospective study of the medical records of patients who presented with symptomatic VTE (deep vein thrombosis [DVT] and/or pulmonary embolism [PE]) concomitant with SARS-CoV-2 infection, treated at Albert Einstein Jewish Hospital, São Paulo, Brazil, between March and July of 2020. This study was approved by the institution’s Ethics Committee under protocol 30809720.9.0000.0071, report number 4.126.297.

The sample only included patients in whom COVID-19 was confirmed by a laboratory test (positive real-time PCR for COVID-19).

In cases in which a physician’s clinical assessment suggested presence of DVT, patients were subjected to a duplex scan of the limb and additional computed pulmonary angiotomography or angiogram to rule out PE. When imaging studies were positive for DVT or PE, patients were included in the study. Clinical suspicion for DVT was defined by pain and/or swelling of one or more limbs, with or without high VTE probability according to the Wells criteria.^12^

For included patients, the incidence of VTE, demographic characteristics, DVT and PE site, DD variation over time, anticoagulation regimen, additional treatment methods, and outcomes were analyzed.

### Statistical analysis

Categorical data are expressed as absolute frequencies and percentages and continuous data are expressed as means with standard deviations and minimum-maximum values. DD values above 3,000 ng/mL were compared according to time of observation using generalized estimating equations (GEE), assuming exchangeable correlations between times. The Bonferroni method was used to identify at which times differences occurred. Tests were performed using a 5% significance level.

## RESULTS

During the period of this study, 484 confirmed cases of COVID-19 were admitted to our service. Sixty-four patients (13.22%) presented some form of clinical suspicion of DVT and underwent a duplex scan.

Thirteen of the patients evaluated (2.68% overall, 20.31% of those subjected to imaging evaluation) presented VTE: there were five cases of concomitant DVT and PE; six cases of isolated DVT; and two cases in which PE was identified but no thrombus was visible in the limbs. For this incidence and sample, the 95% confidence interval ranges from 1.3 to 4.1%; and the precision for this sample is 1.45%.

The population, clinical, and imaging characteristics of all cases of concomitant COVID-19 and symptomatic VTE are presented in Table 1. The average age was 62.7 years, ranging from 40 to 97 years (median 62). Seven patients (53.8%) were male.

**Table 1:** population characteristics and number of preexisting medical conditions.

Baseline Characteristics			
	N	%
***Gender***		
Male (%)	7	53.8
Female (%)	6	46.2
***BMI kg/m^2^(Mean)***		50.7 ± 6.6
<18.5 (%)	1	7.7
18.5-24.9 (%)	2	15.4
25-29.9 (%)	6	46.2
30-34.9 (%)	2	15.4
35-39.9 (%)	1	7.7
> 40 (%)	1	7.7
***Preexisting Conditions***	***N***	***%***
0	2	15.3
1 to 2	2	15.3
>2	9	69.2
***Preexisting Conditions***		
	***N***	***%***
*Dyslipidemia*	*7*	53.8
*SAH*	*5*	38.5
*DM*	*4*	30.8
*Cancer*	*3*	23.1
*Smoking*	*2*	15.4
*Thrombophilia*	*2*	15.4
*COPD*	*1*	7.7
*CAD*	*2*	15.4
*Previous VTE Episode*	*1*	7.7

Over one third of the sample presented two or more preexisting medical conditions, the most common of which was overweight, noted in 76.9% of patients. Other common comorbidities were dyslipidemia (58.8%) and systemic arterial hypertension (SAH) (38.5%).

All of the patients were treated as in-patients. In one case, the patient had been hospitalized for three months prior to manifestation of COVID-19 symptoms, in palliative care due to a neoplasm and advanced age.

Only 1 of the 13 patients had a prior personal history of VTE; two were current smokers and all others denied current smoking habit.

Eleven patients (84.6%) presented with signs suggestive of COVID-19 on the admission CT-scan. In 10 cases, the patients’ initial clinical status was considered severe and they were immediately admitted to an intensive care unit (ICU).

One of the three patients who were initially stable ultimately required transfer to an ICU bed due to clinical deterioration associated with VTE. Although the other two were diagnosed with DVT, they remained stable and did not require respiratory support (one of these was the aforementioned patient in palliative care).

Among the 10 patients in intensive care, seven required orotracheal intubation (63.6%); six needed hemodialysis (54.5%); and one needed extracorporeal membrane oxygenation (9.1%) for respiratory stabilization.

Six of the 11 DVTs diagnosed through duplex scan were in the lower limbs (54.5%) and five were in upper limbs (45.5%). The upper limb DVTs all occurred in the right arm and were all associated with current or previous use of central venous catheters. In the lower limbs, there was one case of DVT in the popliteal vein, two cases in the tibial veins, and three cases in femoral veins. An upper limb DVT was the culprit in just one of the cases of PE that occurred in association with DVT (20%).

Computed tomography angiography was the diagnostic method for six cases out of seven PE patients. In the remaining patient, the diagnosis was made by catheter angiography and echocardiogram. PEs were bilateral in six cases (85.7%). Trunk involvement was observed in one case, segmental involvement in three cases, and the remaining four cases were subsegmental PEs. Only one patient presented with right ventricle overload and pulmonary artery hypertension on echocardiogram.

**Figure 1:**
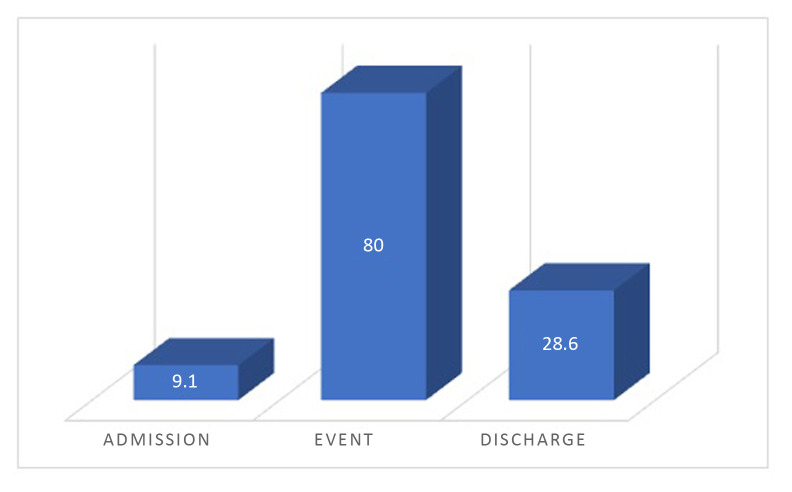
Percentages of patients with D-dimer levels > 3000 ng/mL at admission, on the day attributed to VTE onset, and after acute VTE resolution.

It was possible to analyze DD variation over the course of the hospital stay in 11 of the 13 cases, which are illustrated in Figure 1. Only three (27.2%) of the patients analyzed had DD levels below the reference values (500 ng/mL) at admission and only one patient had levels exceeding 3,000 at admission – in this case, DVT was already present.

We found that DD levels were above 3,000 ng/mL on the day attributed to VTE onset in 8 (80%) patients and that these levels were statistically higher than the baseline admission levels (*p* < 0.05). On the day of hospital discharge, a statistically significant decrease was also observed in DD values (*p* < 0.05).

In this series, all patients (100%) received pharmacological VTE prophylaxis or full anticoagulation, starting at admission. The institution’s choice of pharmacological prophylaxis is low molecular weight heparin (LMWH) at 40 mg once daily, unless contraindicated. When renal function precludes use of LMWH, unfractionated heparin (UFH) is employed. Mechanical prophylaxis (standard anti-embolism 13-18mmHg stockings or intermittent pneumatic compression devices) is administered in association with an anticoagulant for all critical patients. Mechanical prophylaxis is used in isolation when pharmacological prophylaxis is contraindicated.

In four cases (30.7%), patients received full dose anticoagulation starting at admission because VTE was diagnosed at the first examination. In all other cases (69.3%), patients were started on a prophylactic regimen at admission, until a confirmed VTE diagnosis provoked dosage change.

Mechanical prophylaxis was used as an adjuvant method in 11 patients (84.6%), all of them critical: 10 in intensive care and 1 in the palliative care ward. In two cases, compressive stockings were used in isolation and in 9 cases in association with intermittent pneumatic compression devices.

Full anticoagulation therapy was started immediately after confirmation of VTE by imaging for 12 patients (4 at admission and 8 later on during hospitalization). LMWH is the institution’s drug of choice and was used in seven patients; five patients were given UFH due to renal impairment. One patient, who was in palliative care for an advanced neoplasm, was maintained on a prophylactic regimen.

Complications related to anticoagulation were observed in 4 of the 12 treated patients, with one type 1 complication (hematuria) and three type 2 complications (two cases of minor gastrointestinal bleeding and one case of hemoptysis), according to the Bleeding Academic Research Consortium definitions.

In one case, mechanical thrombectomy associated with thrombolysis was required due to massive PE. The procedure was successful and the patient was extubated 10 days after the procedure, with no signs of permanent clinical damage and unaltered echocardiogram at discharge.

The mean hospital stay was 33.4 days (ranging from 2 to 49 days). In patients in whom a VTE diagnosis was not present at first examination, median time from admission to VTE diagnosis was 14 days (ranging from 4 to 82 days). In-hospital VTE occurred in only one case in a ward setting and this was the palliative care patient. One patient presenting concomitant COVID-19 and DVT at admission remained stable throughout treatment with no need for intensive care. In all other cases, either patients were admitted directly to the ICU with DVT or DVT occurred while in the ICU.

Two deaths occurred during the study, both of patients who had severe comorbidities: one patient with advanced stage neoplasm and a second patient who had recently undergone cardiac surgery complicated by an infection. In one case of in-hospital death (50%), the patient could not be given anticoagulation therapy due to an advanced neoplasm.

At the end of our study protocol, nine patients had been discharged and two remained hospitalized, but had no signs of worsening of their condition due to the VTE event, including the patient in palliative care. All nine discharged patients received oral anticoagulation for secondary VTE prophylaxis, as prescribed by their attending physicians: six of them received rivaroxaban, two received warfarin, and one received dabigatran.

## DISCUSSION

In this series, out of 484 in-patients hospitalized due to COVID-19 infection in the clinical ward (stable patients) or in the ICU (severe patients), sixty-four (13.22%) presented symptoms leading to investigation of DVT. Of these, 13 (20.31%; 2.7% overall) presented a confirmed case of VTE.

It has been well established that moderate to severe presentations of the SARS-CoV-2 infection may present with acute renal failure, severe hypoxia, and cardiac arrhythmias.^13^ The virus can cause damage through widespread pro-inflammatory cytokine responses, increased procoagulant factors leading to coagulation chain disorder and elevation of fibrin degradation products, thus raising DD levels.^6^

Based on this premise, VTE risks associated with COVID-19 have been fiercely discussed, with an assumption that these patients were exposed to increased risk of thromboembolic events. Several studies were conducted assessing the VTE prevalence in COVID-19 patients: most of them reported an incidence of over 20%.^7^^,^ 8 ^,^ 10 ^,^ 14 ^-^ 19 However, one study, conducted in China by Xu et al.,^11^ found that 2.9% of COVID-19 patients developed VTE.

Expert consensus, however, still suggests against routine ultrasound screening for detection of asymptomatic DVT, even in critically ill COVID-19 patients.^20^ In the recent CHEST Guideline and Expert Panel Report it was noted that, although routine ultrasound screening is not recommended, clinicians ought to have a low threshold for imaging investigation with a reasonable degree of clinical suspicion for VTE.^20^ For this reason, at our institution, patients with COVID-19 were investigated even if Wells criteria were suggestive of low probability.

In four patients with severe disease, VTE was diagnosed immediately and they were directly referred to the ICU because of the diagnosis of simultaneous COVID-19 and VTE. The other nine patients were hospitalized with COVID-19 and developed VTE over the course of their hospital stays. In accordance with previous studies, most of our patients (84.6%) developed VTE while in intensive care. Only two patients in our series were diagnosed with VTE while stable and in clinical wards. This predominance of VTE cases occurring in ICUs has been repeatedly observed in several series in the literature.^7^^-^ 9

The most frequent VTE risk factor in this series was obesity and overweight, noted in 76.9% of patients, which is also common in patients without COVID-19.^21^ Most patients presented two or more comorbidities, of which dyslipidemia, SAH, and diabetes mellitus (DM) were the most common. These comorbidities are well-established factors for worsening of SARS-CoV-2 infections and have a negative effect on patients’ prognoses. Furthermore, COVID-19 may worsen preexisting heart conditions.^9^^,^ 21

With respect to DVT site, DVT was more frequent in the lower limbs. When DVT did occur in the upper limbs, it was invariably associated with current or previous use of central venous catheters and only one upper-limb DVT complicated with PE. The diminished incidence of PE in association with upper-limb DVT is in accordance with general population data.^22^ In a study published by Yamashita et al.,^23^ the global prevalence of DVT in upper limbs was 3%, with 58% of these patients having been diagnosed with cancer and 22% being on central venous catheters, with a 14% rate of associated PE.^23^

Since Lippi et al.^24^ published an analysis that demonstrated a 2.5 fold increase in the DD values in patients with severe COVID-19, papers regarding this marker have abounded, including those proposing DD levels over 2,000 at hospital admission as an effective predictor of mortality.^25^ However, these findings are questionable, especially with regard to the difficulty of standardizing these tests.^26^ For this reason, our institution chose not to define the necessity of anticoagulation based solely on laboratory findings. Prophylaxis was offered on the same basis as for routine clinical inpatients, unless there were contraindications.

It has been established that a systematic in-hospital VTE prophylactic approach can greatly diminish the occurrence of thromboembolism.^27^^,^ 28 However, some studies suggest high prevalence of VTE in COVID-19 patients despite adequate prophylaxis.^15^^,^ 19 Even though our study is limited to patients who presented clinical symptoms, the prevalence of VTE events is far lower than that reported by recent studies. In one study of 138 consecutively enrolled COVID-19 patients, only 2.9% (4 patients) were effectively diagnosed with VTE, even though 16.67% were classified as high-risk according to the Padua Prediction Score.^11^ The authors attributed these findings to a consequence of effective thromboprophylaxis in patients classified as being at high risk of thrombosis.^11^

Only one patient presented with massive PE. He was a morbidly obese patient who was promptly treated by catheter directed thrombectomy and thrombolysis after presumptive PE diagnosis through an altered echocardiogram combined with a duplex scan finding of DVT. This patient had satisfactory progress and was discharged in good condition. The determination for early intervention as opposed to systemic thrombolysis is well endorsed in current literature, especially considering that the patient was at high risk for bleeding events.^29^^,^ 30

Only two deaths occurred from COVID-19 associated with VTE during the observation period. Both patients had severe comorbidities: one with advanced stage neoplasm and a second patient in recent recovery from cardiac surgery complicated with infection. Two patients over 90 years of age survived, demonstrating that these cases ought to be addressed individually and without prejudice.

This study is limited primarily by being a single center retrospective analysis, and also due to the fact that protocols for management of COVID-19 patients are not yet fully established, with lack of conduct standardization since this is not a totally elucidated disease.

Further multicenter research is needed for better understanding of the physiopathology of the association between COVID-19 and VTE,^31^^,^ 32 enabling more accurate management and leading to better survival outcomes.

## CONCLUSION

Overall symptomatic VTE prevalence in 484 hospitalized COVID-19 patients was 2.7%, accounting for to 20.31% of patients who underwent imaging investigation after clinical suspicion. Thromboembolic events were more frequent in patients in intensive care.

Early institution of prophylaxis and immediate full anticoagulation when VTE is diagnosed should be the key goals for those who treat this type of patient.
